# Recurrent invasive meningococcal infections – quantifying the risk, Germany, 2002 to 2018

**DOI:** 10.2807/1560-7917.ES.2020.25.25.1900565

**Published:** 2020-06-25

**Authors:** Manuel Krone, Thiên-Trí Lâm, Heike Claus, Ulrich Vogel

**Affiliations:** 1 Institute for Hygiene and Microbiology, National Reference Laboratory for Meningococci and *Haemophilus influenzae*, University of Würzburg, Würzburg, Germany

**Keywords:** recurrent infections, invasive meningococcal disease, complement deficiency, prevention

## Abstract

**Introduction:**

Invasive meningococcal disease (IMD) is a rare condition with a high case fatality rate. While most patients suffer from one single episode in life, there is anecdotal evidence for recurrent infection.

**Aim:**

The German National Reference Laboratory for Meningococci and *Haemophilus influenzae* (NRZMHi) analysed IMD cases from 2002 to 2018 to retrospectively quantify the risk of recurrent infection.

**Methods:**

Recurrent IMD was defined as detection of *Neisseria meningitidis* in a sample of the same patient more than 30 days after the first episode of IMD.

**Results:**

Among 5,854 patients with a median observation period of 9.4 years, 14 suffered a second IMD episode and one patient a third one. The risk of a recurrent IMD was 29.4 per 100,000 person-years for survivors of the first episode. Rare serogroups (Y, W, E and Z) were more common in patients with recurrent IMD (p < 0.0001).

**Discussion:**

Patients surviving IMD were at least at a 50-fold risk of another IMD episode compared with the general population. The study most likely underestimated the risk of recurrent infection. Increased risk may be due to undiagnosed complement deficiencies. The high risk of re-infection argues for vaccination of patients who have survived IMD.

## Introduction

Invasive meningococcal disease (IMD) is a rare condition (1.2 million cases per year worldwide, ca 15–20 cases per 100,000 population) with a high case fatality rate (worldwide more than 10%) [1]. While most affected patients suffer from only a single episode in their life span, there is anecdotal evidence for recurrent infection [2-4]. It is known that the prevalence of complement deficiencies is especially high in patients with recurrent IMD [5-7] but there are also reports of recurrent IMD associated with Waldenström’s disease [2], chronic glomerulonephritis [3] and IgG_2_-subclass deficiency [4]. Host predisposition inside and outside the complement system seems to play an important role in the aetiology of IMD [8,9].

Previous data suggest that rare serogroups are more frequent among recurrent IMD patients and that the course of disease is less severe [6,10]. The proportion of female patients is reported to be lower [6]. The incidence of recurrent IMD, however, has not been quantified.

Knowledge of the frequency of recurrent disease informs decisions on vaccination of reconvalescent cases as well as the use of diagnostic tests to assess complement deficiency.

The Centers for Disease Control and Prevention recommend vaccination for all preteen and teen IMD cases in the United States (US) [11]. The Spanish Association of Paediatrics recommends a vaccination against serogroup ACWY meningococci (MenACWY) for all persons with history of previous IMD [12]. Public Health England recommends IMD patients to catch up the generally recommended meningococcal vaccinations but no additional vaccination [13]. There are no vaccination recommendations for IMD survivors in Germany [14], France [15], Italy [16], the Netherlands [17], Sweden [18] and Switzerland [19], nor in the guidance document on IMD from the European Centre for Disease Prevention and Control [20].

In Germany, vaccination against MenC is recommended at the age of 1 year. Beyond this age, persons with congenital or acquired immunodeficiencies, e.g. complement deficiencies, are recommended MenB as well as MenACWY immunisation [14]. It is recommended that close contacts of IMD patients receive post-exposure prophylaxis (ciprofloxacin, rifampicin or ceftriaxone) [21]. Since 2009, vaccination of household contacts of an IMD case with either a MenC or a MenACWY vaccine has been recommended depending on the serogroup of the index case [22]. Since 2015, this recommendation has been extended to serogroup B [23].

The aim of this retrospective observational study was to determine the incidence of recurrent IMD.

## Methods

### Study population

We analysed IMD cases reported to the German National Reference Laboratory for Meningococci and *Haemophilus influenzae* (NRZMHi) between 2002 and 2018 as part of the laboratory surveillance programme. Submissions had to meet the European Union (EU) case definition of IMD, i.e. isolation or detection of *Neisseria meningitidis* from a normally sterile site or purpuric skin lesions [24], and had to be allocated to specific patients. Only German residents were included in the study.

### Strain typing

The serogroup of all samples was determined by slide agglutination using Remel agglutination sera or by serogroup-specific PCR. The *N. meningitidis*-specific genes *porA* and *fetA* were amplified by PCR and subsequently sequenced. Finetypes were defined by the combination of serogoup, *porA* variable region (VR)1, *porA* VR2 and *fetA* VR [25]. Only samples with a complete finetype were included in the strain typing analysis.

### Identification of recurrent invasive meningococcal disease patients

The NRZMHi receives patients´ samples and meningococcal isolates for species identification, antimicrobial resistance testing and typing. Upon submission, samples are assigned to patients’ identification numbers (ID) based on information from the NRZMHi submission form. Samples with an identical patient ID were grouped. By applying a threshold of 30 days between the submission dates, probable recurrent infections were identified.

### Modelling

**Figure 1:**
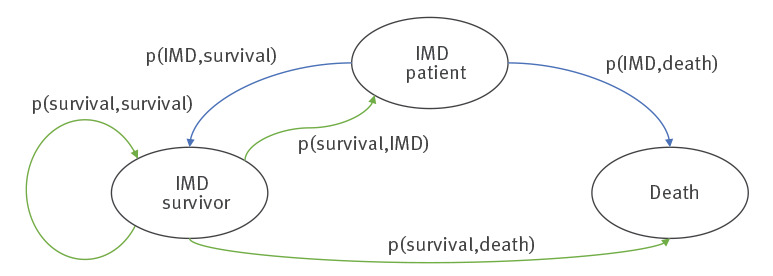
States of the Markov model for recurrent invasive meningococcal infections, Germany, 2002 to 2018

A Markov model was created to model the cohort of IMD patients during the observation period to estimate population dynamics with steps representing 1 calendar year (Figure 1). This modelling was necessary as survival data of the patients were not available.

Blue: transitions in the first year after IMD; green: transitions in consecutive years.

IMD: invasive meningococcal disease; p(IMD,death): probability to die from IMD; p(IMD,survival): probability to survive IMD; p(survival,death): probability to die due to another cause of death; p(survival,IMD): probability of a recurrent IMD; p(survival,survival): probability to neither suffer from a recurrent IMD nor die from another cause.

We used the average IMD case fatality rate of 9.6% from 2012 to 2015 in Germany to estimate the risk of dying within 1 year after IMD [26]. In consecutive years, the mortality of the individuals in the cohort was estimated applying general German mortality tables for the simulated year and the individual year of birth [27]. A Monte Carlo method with 1,000 runs was used to determine the number of deaths and observation years.

The model was implemented in Microsoft Excel 2016.

### Number needed to vaccinate

The number needed to vaccinate (NNV) in order to prevent one case in the cohort of surviving IMD patients was calculated using the following formula [28]:

$$NNV\;=\;\frac{100,000}{\left(IMD\;cases\;per\;100,000\;person–years\right)\times \left(Vaccine\;effectiveness\right)\times \left(Strain\;coverage\right)}$$

Vaccine effectiveness (VE) was estimated at 80% as a relevant proportion of the recurrent IMD patients may have detected or undetected complement deficiencies [23].

Strain coverage (SC) was calculated using the serogroup (SG) distribution of recurrent IMD and the vaccine coverage, which was assumed to be 100% for serogroups A, C, W and Y as MenACWY vaccines target the capsular polysaccharides. For MenB vaccines, which target surface antigens, the coverage was assumed to be 82% [29]. Total SC was calculated summing up the proportions of the single serogroups in the recurrent IMD population multiplied by the estimated vaccine coverage for this serogroup:

$$SC=\;\sum_{\forall SG}proportion\;of\;SG\;\times vaccine\;coverage\;of\;SG$$

### Vaccination costs

Vaccination costs were calculated using German vaccine prices acquired from the mobile app Arznei aktuell [30]: EUR 108.34 for one dose of either MenB vaccine (Bexsero or Trumenba) and EUR 52.69 for MenACWY vaccines as the mean between the costs for one dose of Menveo (EUR 54.66) and Nimenrix (EUR 50.72) [30]. The administration cost of EUR 8.00 per vaccinated dose was taken from a vaccination agreement for patients with general insurance in Bavaria [31].

### Costs of complement deficiency screening

We calculated a complement deficiency screening scenario assuming that every IMD patient gets a CH50 (classical pathway) and an AH50 (alternative pathway) test as screening for complement deficiencies. The price was acquired from the German tariff for doctors list [32]: EUR 29.14 for CH50 and EUR 34.97 for AH50, summing up to EUR 64.11. For the model, we assumed that 1% of the IMD patients had a complement deficiency according to the most extensive dataset on this topic from Platonov et al. [6]. Quantitative and functional testing of on average five single factors of the complement system summed up to EUR 145.70 for IMD patients with abnormal CH50 or AH50 [32]. In this scenario, IMD patients who tested positive for complement deficiency were vaccinated as described above. Additional personnel costs were estimated as EUR 20 for the basic complement screening and EUR 200 for the detailed complement analysis.

According to Platonov et al., it can be assumed that complement deficiencies could be detected in 50% of the recurrent IMD patients [6].

### Statistics

Two-tailed Fisher’s exact test was used to compare sex, frequency of serogroups and frequency of finetypes in recurrent IMD patients with other IMD patients. The Mann–Whitney U test was used to compare age between the two groups. IBM SPSS Statistics 25 was used for the statistical analysis.

### Sensitivity analysis

As the Markov model simulates only high-quality evidence of recurrent IMD, sensitivity analyses were performed to demonstrate the effect of uncertainty factors on the results. Univariate analyses were performed for the two variables ‘underdiagnosis and underreporting’ and ‘laboratory surveillance’.

Azzari et al. estimated by comparing surveillance data with hospital discharge records in Italy that around 70% of IMD cases were reported to the health authorities but even within one country, big regional differences in under-reporting could be shown [33]. This value was used as an approximation for underdiagnosis and under-reporting of IMD in the sensitivity analysis.

Reporting of IMD cases is mandatory in Germany but submitting samples to the NRZMHi is voluntary [34]. The laboratory surveillance rate was determined by the quotient of the IMD cases included in the study divided by the number of IMD cases reported to the Robert Koch Institute (RKI), the German national health authority [35]. While 5,869 IMD cases were included in the study, 7,448 cases of IMD were reported to the RKI in the same period, resulting in a laboratory surveillance coverage of 79% [35]. This value was used in the sensitivity analysis as laboratory surveillance rate. As all NRZMHi-confirmed cases are merged and included in the RKI database, there is only risk for an underestimation of the rate of recurrent IMD but not for overestimation.

### Multivariate probabilistic model

In the multivariate probabilistic model the influence of underdiagnosis, under-reporting and laboratory surveillance coverage was included. Underdiagnosis and under-reporting were added as uniformly distributed variables between 70% and 100%. The laboratory surveillance coverage was simulated as a uniformly distributed variable between the laboratory surveillance rate and 100%. In addition, the multivariate probabilistic model was run as Monte Carlo method with 1,000 iterations.

### Ethical statement

Ethical approval was not required for this study as analysis of the pseudonymised data of IMD cases is one of the official functions of the NRZMHi.

## Results

### Study population

**Figure 2:**
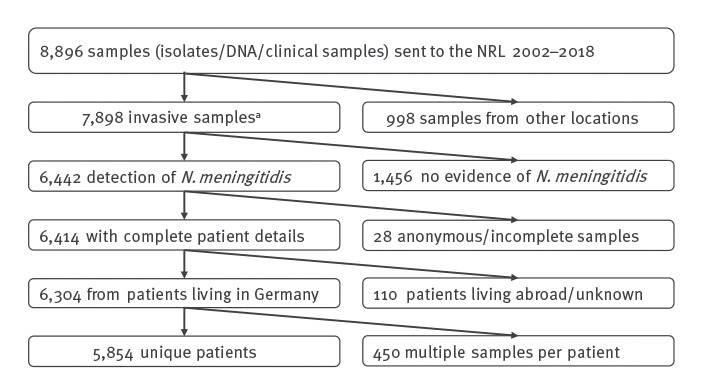
Selection of the study population, recurrent invasive meningococcal infections, Germany, 2002 to 2018 (n = 8,896)

Of 8,896 submissions to the NRZMHi, 998 were excluded as the cases did not meet the EU case definition. Furthermore, 1,456 samples did not show evidence for *N. meningitidis*: 1,178 patients’ samples were PCR-negative for *N. meningitidis*, 246 strains did not grow and in 32 cases, the species *N. meningitidis* was not confirmed. Twenty-eight samples could not be assigned to patients. Consequently, 110 patients living abroad or with unknown place of residence were excluded. In sum, 6,304 samples remained that met the inclusion criteria of the study (Figure 2).

NRL: national reference laboratory.

^a^ According to the European Union case definition [24].

We identified 5,854 unique patients, resulting in 450 samples that were not unique. Among the 450, 435 were assumed duplicates submitted within the threshold of 30 days between submitting days; they had the same finetype as the primary submissions and were thus excluded from further analysis. By contrast, the remaining 15 submissions were received from the presumed same patient more than 30 days after the primary submission betrayed different finetypes and were therefore considered as recurrent IMD and included in the further analysis.

### Recurrent invasive meningococcal disease patients

The observation period for single patients in the cohort after their first IMD episode until 31 December 2018 ranged between 0 and 16.9 years (median: 9.4 years; IQR: 3.9–13.7 years). Among the 5,854 patients, 13 suffered two episodes. One person had three infections (Table). The median interval from the first to the second episode was 19.9 months (IQR: 11.7–36.1 months).

**Table:** Cases of recurrent invasive meningococcal disease, Germany, 2002 to 2018 (n = 14)

Case	Age at IMD1 (year)	Interval to IMD2/3 (year)	Finetype IMD 1	Finetype IMD 2/3
1	3 months (2016)	10 months (2017)	B:P1.12–1,13–6:F5–2	B:P1.12–1,13–32:F5–2
2	4 months (2007)	6 months (2007)	B:P1.7–1,1:F3–6	B:P1.7–2,4:F1–5
3	11 years (2002)	5 years 9 months (2008)	C:P1.5–1,10–6:F3–6	B:P1.17,9:F1–7
4	14 years (2008)	1 year 7 months (2010)	Y:P1.5–2,10–1:F4–29	W:P1.18–1,3:F4–1
5	14 years (2012)	1 year 10 months (2014) 3 years 3 months (2015)	Y:P1.5–2,10–1:F4–1	W:P1.18–1,3:F4–1 B:P1.22,14:F5–5
6	15 years (2016)	7 months (2016)	NG:P1.22–11,15–25:F5–1	B:P1.12–1,13:F1–5
7	16 years (2002)	3 years 2 months (2005)	Y:P1.5–1,10–6:F3–6	C:P1.5–1,2–2:F3–3
8	16 years (2010)	1 year 5 months (2011)	Y:P1.5–1,10–4:Fn.d.^a^	B:P1.5–1,10–4:F1–5
9	17 years (2006)	11 months (2007)	W:P1.5,2:F:4–1	W:P1.18–1,3:F4–1
10	17 years (2005)	1 year (2006)	B:P1.17,9:F1–7	Y:P1.5–2,10–1:F4–1
11	18 years (2016)	2 years 5 months (2018)	Y:P1.5–2,10–1:F4–1	NG:P1.19,15:F5–1
12	19 years (2008)	1 year 8 months (2009)	B:P1.12–1,16:F4–3	B:P1.17,9:F1–7
13	20 years (2003)	7 years 3 months (2010)	E:P1.5,2:F1–7	Z:P1.18–1,3:F2–9
14	20 years (2010)	6 years 8 months (2017)	B:P1.12–1,13–1:F1–7	B:P1.17–1,23–13:F1–5

IMD: Invasive meningococcal disease; IMD1/2/3: IMD first/second/third episode; NG: non-groupable.

^a^ Not determined, a stop-codon was found inside the *fet*A-gene.

There were no statistically significant differences between recurrent IMD patients and the patients with only one IMD episode regarding median age (16.4 years; IQR: 14.0–18.7 years vs 16.3 years; IQR: 2.3–29.4 years; p = 0.74). Not statistically significant, there were fewer females cases among recurrent IMD patients (28%; 4/14 vs 48%; 2,786/5,840; p = 0.19) (Table).

### Strain typing

**Figure 3:**
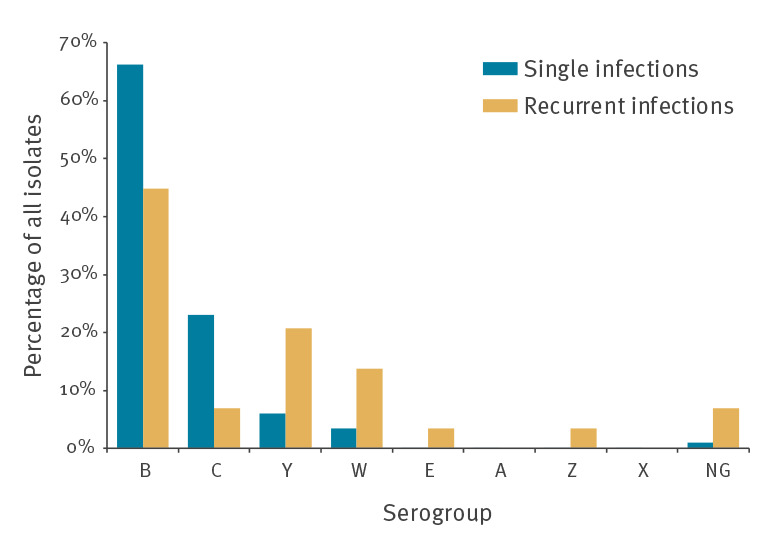
Serogroup distribution of single and recurrent invasive meningococcal infections, Germany, 2002 to 2018 (n = 5,869)

The serogroups of the 29 strains (14 from first episodes, 14 from second episodes, one from a third episode) isolated from the 14 patients with recurrent infections were compared with 5,840 strains from the reference cohort. Serogroups Y (21%; 6/29 vs 6%; 351/5,840; p = 0.0070), W (14%; 4/29 vs 4% 205/5,840; p = 0.023), E (3%; 1/29 vs 0.14% 8/5,840; p = 0.044), Z (3%; 1/29 vs 0.03%; 2/5,840; p = 0.015) as well as non-groupable meningococci (7%; 2/29 vs 1%; 56/5,840; p = 0.033) were significantly overrepresented. Shares of the generally most common serogroups B (45%; 13/29 vs 66%; 3,867/5,840; p = 0.018) and C (7%; 2/29 vs 23%; 1,345/5,840; p = 0.044) were significantly lower than average (Figure 3). None of the recurrent IMD was caused by meningococci of the serogroups A and X which are extremely rare in Germany.

NG: non-groupable.

The complete finetype could be determined for 28 of 29 samples from recurrent infections (Table) and for 5,550 of 5,840 samples from single infections. A premature stop codon was found in the *fet*A gene of one isolate from a recurrent IMD patient. A total of 22 different finetypes were detected in recurrent infections, including three finetypes occurring three times each. The same strain was not observed more than once in a patient, although once (Patient 1) both strains shared *porA* VR1 and the *fetA* allele. The sequence type of both strains of this patient turned out to be identical. 

**Figure 4:**
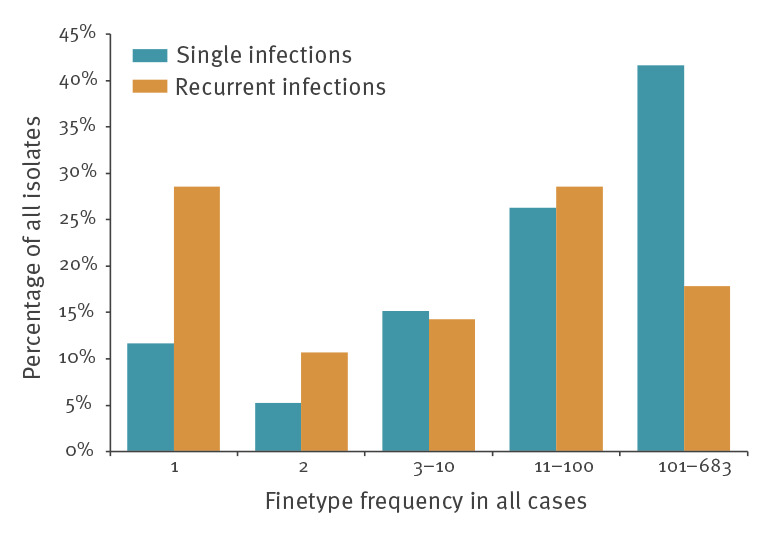
Finetype distribution in single and recurrent invasive meningococcal infections, Germany, 2002 to 2018 (n = 5,578)

Unique finetypes in the cohort of German IMD patients were significantly more common in recurrent IMD patients compared with other IMD patients (29%; 8/28 vs 12%; 646/5,550; p = 0.013) while finetypes occurring more than 100 times were markedly less common in recurrent IMD patients (18%; 5/28 vs 42%; 2,312/5,550; p = 0.012) (Figure 4).

### Risk estimation

Cumulatively, 57,098 person-years (py) were observed in the modelled cohort. In the simulated cohort, on average 12.9% (755/5,854; IQR: 737–770) of cases died during the observation period, thereof 562 (IQR: 545–578) deaths were due to IMD. This resulted in 51,024 py lived (IQR: 50,866–51,200 py lived).

The overall incidence of a recurrent IMD was 29.4 (IQR: 29.3–29.5) per 100,000 py of survivors of the first episode with highest values in the first (74.8 per 100,000 py) and second (101.2 per 100,000 py) year after the initial IMD. Compared with an average general incidence of IMD of 0.56 per 100,000 py in the observation period [35], the relative risk (RR) for recurrent IMD was 52.5 (IQR: 52.3–52.7).

### Sensitivity analysis

Analysing the uncertainty factor of underdiagnosis and under-reporting in a univariate sensitivity analysis and assuming that 70% of the IMD cases were diagnosed and reported correctly, the incidence of recurrent IMD rose to 42.0 per 100,000 py, and the RR rose to 75.0. Extrapolating the laboratory surveillance coverage in Germany of 79% as a single uncertainty factor, the incidence of recurrent IMD increased to 37.2 per 100,000 py, and the RR increased to 66.4. Combining assumptions on underdiagnosis, under-reporting and the laboratory surveillance rate, the incidence of recurrent IMD was 53.2 per 100,000 py, and the RR was 94.9.

In the multivariate sensitivity model performed to analyse the combined effect of underdiagnosis, under-reporting and the laboratory surveillance rate distributed stochastically, the median incidence of recurrent IMD was 39.1 (IQR: 35.2–43.1) per 100,000 py, and the RR was 69.7 (IQR: 62.9–76.9).

### Number needed to vaccinate

Strain coverage of the recurrent IMD strains by MenB and MenACWY vaccines was calculated as follows:

SC = 45% × 82% (B) + 7% × 100% (C) + 14% × 100% (W) + 21% × 100% (Y) = 78%

Using data from the conservative estimation and under the conservative assumption that the duration of vaccine protection was 2 years, the number needed to vaccinate to prevent one case in the first year after the initial IMD episode was:

$$NNV\;=\;\frac{100,000}{\left(Incidence\;in\;the\;first\;2\;years\;per\;100,000\right)\times \left(VE\right)\times \left(SC\right)}=\frac{100,000}{\left(74.8+101.2\right)\times 80\%\times 78\%}=909$$

### Vaccination costs

The vaccination costs per person included two doses of a MenB vaccine, one dose of a MenACWY vaccine and three times the administration costs of the vaccine, amounting to EUR 293.37 per vaccinated patient for the basic immunisation based on German prices. Potential revaccinations would lead to additional costs.

Assuming a vaccination protection of 2 years after the initial IMD episode, this resulted in EUR 266,673.33 per prevented IMD case over a 2-year period after vaccination.

### Costs of complement deficiency screening

Screening all IMD patients for complement deficiencies to vaccinate only patients with such deficiencies resulted in costs for the basic screening of EUR 84.11 and an additional EUR 639.07 for the 1% with abnormalities in the screening, including vaccination costs. Thus, the average cost per IMD patient summed up to EUR 90.50. As complement deficiencies are detected in half of the recurrent IMD cases [6], the number needed to screen to prevent one case of recurrent IMD by vaccination doubled to 1,818. The complement deficiency screening scenario resulted in EUR 164,530.27 per prevented IMD case. This scenario did not take into account IMD cases without detectable complement deficiency.

## Discussion

Patients surviving IMD are at a more than 50-fold increased risk of another IMD episode compared with the general population, estimated in a conservative simulation that most likely underestimated the risk of recurrent infections. Considering underdiagnosis, under-reporting, and the laboratory surveillance coverage, the real risk was increased ca 70-fold. These data rebut the proposition that IMD survivors are protected by antibodies [15,17].

Increased risk may be caused by diagnosed and undiagnosed complement deficiencies or other congenital or acquired immunodeficiencies [5,6]. Consistent with the literature [6], recurrent IMD was more common in male than in female cases, even though the sex difference was not significant in our setting.

In 13 of the 14 patients, the second or third IMD episode was caused by strains differing from the first episode. However, in Patient 1, where the strains of both episodes shared the same serogroup, *porA* VR1, *fetA* and sequence type, it cannot be excluded that the strain circulated in contact persons, adopted a mutation of *porA* VR2 over time and affected the patient again.

The estimated risk of IMD survivors to suffer from recurrent IMD episodes is at least 2.5 times higher than the incidence of 11 per 100,000 py estimated for asplenic patients or patients with splenectomy to suffer from IMD [23].

Our study had limitations. The risk of IMD in general and the particular risk of recurrent IMD may be underestimated because of underdiagnosing and under-reporting of IMD cases. A sensitivity analysis was performed to demonstrate the effect on the results. It remains unclear if the estimation by Azzari et al. that we used in the sensitivity analysis, i.e. that around 70% of IMD cases in Italy are reported to the health authorities, is applicable to Germany [33].

It is also unclear if the data on complement deficiencies in IMD patients with one or more episodes in Russia, raised by Platonov et al. [6], are transferable to the German setting as the prevalence of complement deficiencies in the population differs between countries [5]. Furthermore, the meningococcal strain composition was unique in the cohorts analysed by Platonov et al., with a high prevalence of serogroup A.

The rate of underdiagnosis may even be higher in patients with complement deficiencies as they may be under antibiotic prophylaxis [36]. IMD in these patients may be underdiagnosed owing to the effect of the antibiotics on the meningococci in blood or cerebrospinal fluid before samples are taken for microbiological diagnostics.

As only 79% of the reported cases could be assigned to samples in the NRZMHi, it can be assumed that also recurrent IMD infections were missed because of failure to submit to the NRZMHi. This factor was included in the sensitivity analysis. Data can be missing for several reasons: In some cases, samples were not submitted at all for typing at the NRZMHi. Some submissions lacked information about the patient or data were incomplete for other reasons. In a few submitted isolates, typing at the NRZMHi failed because the strain could not be recultivated and PCR was unsuccessful. Assuming that recurrent IMD samples were sent to the NRZMHi as often as samples from a first episode, the real incidence of recurrent IMD may be underestimated, too. However, since a recurrent infection psychologically may seem more relevant, submissions of these cases are more likely to be submitted and registered by the NRZMHi laboratory surveillance than other IMD.

The NRZMHi had no access to medical records of the IMD patients. Incomplete data hampered the identification of recurrent infections. Thus, the incidence of recurrent IMD may have been underestimated.

Because patient data were lacking, general IMD case fatality was used to calculate the risk of death. There is evidence that recurrent IMD courses are less severe than other IMD [6]. As only incomplete case fatality data were communicated to the NRZMHi and numbers were low, it remains unclear if case fatality of recurrent IMD patients corresponds to the general IMD case fatality.

It may be speculated if survivors of an IMD episode have the same mortality in the years after the IMD as the general population or if the mortality is increased. Sequelae of the IMD episode may lead to an increase. Also underlying diseases, e.g. complement deficiencies, may result in both an increased risk for IMD and an increased mortality by other causes, e.g. other infections or autoimmune disorders.

## Conclusion

The high risk of recurrent meningococcal infections argues for vaccination of IMD patients following convalescence, especially as their estimated risk is higher than that of e.g. asplenic patients who are recommended to be vaccinated against meningococci of serogroups A, C, W, Y as well as B [37]. To prevent one recurrent case in the first 2 years after a timely carried out vaccination, 909 IMD cases have to be vaccinated. The number is lower than the NNV for asplenic or HIV-positive patients [23]. This justifies a vaccination recommendation even if vaccination costs are high. The costs to vaccinate only those individuals with complement deficiency is only 62% of the cost for vaccinating all patients who survived IMD. However, this approach would only address individuals with detectable complement deficiency. Furthermore, conducting complement deficiency testing first may result in significant delays. As recurrent cases have occurred already some months after the first episode, the vaccination should be given as soon as possible after convalescence.

At least MenACWY vaccines induce antibody response also in complement-deficient patients [10,38-41]. As complement deficiencies are inherited as an autosomal codominant trait [5], it would be reasonable to screen family members of IMD patients with confirmed complement deficiencies. Molecular data of IMD patients in the recent years have shown genetic predispositions inside and outside the complement system [8,9]. The relevance of these findings for the prevention of recurrent IMD has yet to be determined.

An alternative strategy, which could probably prevent a proportion of recurrent IMD cases at much lower cost, is to offer vaccination and complement deficiency screening at least to IMD patients after a first episode with IMD caused by rare serogroups (E, W, Y, Z and non-groupable) which are linked to recurrent IMD and complement deficiencies. The disadvantage of this strategy is that using data from this analysis, more than 40% of recurring IMD patients would be missed.
